# Supply-Side Barriers to Maternity-Care in India: A Facility-Based Analysis

**DOI:** 10.1371/journal.pone.0103927

**Published:** 2014-08-05

**Authors:** Santosh Kumar, Emily Dansereau

**Affiliations:** 1 Department of Economics & International Business, Sam Houston State University, Huntsville, Texas, United States of America; 2 Institute for Health Metrics and Evaluation, University of Washington, Seattle, Washington, United States of America; University of Alabama at Birmingham, United States of America

## Abstract

**Background:**

Health facilities in many low- and middle-income countries face several types of barriers in delivering quality health services. Availability of resources at the facility may significantly affect the volume and quality of services provided. This study investigates the effect of supply-side determinants of maternity-care provision in India.

**Methods:**

Health facility data from the District-Level Household Survey collected in 2007–2008 were analyzed to explore the effects of supply-side factors on the volume of delivery care provided at Indian health facilities. A negative binomial regression model was applied to the data due to the count and over-dispersion property of the outcome variable (number of deliveries performed at the facility).

**Results:**

Availability of a labor room (Incidence Rate Ratio [IRR]: 1.81; 95% Confidence Interval [CI]: 1.68–1.95) and facility opening hours (IRR: 1.43; CI: 1.35–1.51) were the most significant predictors of the volume of delivery care at the health facilities. Medical and paramedical staff were found to be positively associated with institutional deliveries. The volume of deliveries was also higher if adequate beds, essential obstetric drugs, medical equipment, electricity, and communication infrastructures were available at the facility. Findings were robust to the inclusion of facility's catchment area population and district-level education, health insurance coverage, religion, wealth, and fertility. Separate analyses were performed for facilities with and without a labor room and results were qualitatively similar across these two types of facilities.

**Conclusions:**

Our study highlights the importance of supply-side barriers to maternity-care India. To meet Millennium Development Goals 4 and 5, policymakers should make additional investments in improving the availability of medical drugs and equipment at primary health centers (PHCs) in India.

## Background

Policymakers in many developing countries are grappling with low utilization of health services and poor health outcomes, with India as no exception. The World Bank estimates that India is one of the highest ranked countries in the world for malnourishment among young children, child mortality, maternal mortality, and other pregnancy-related complications [1]. India contributes to one-fifth of the global burden of absolute maternal deaths despite experiencing a sustained decline in maternal mortality in the last two decades [2]. The recent improvement in the maternal mortality ratio has been supported by the Government of India's efforts to improve financial and geographic access to care, including the establishment of a wide network of public health facilities and implementation of a variety of outreach and incentive-based programs [3]. In particular, the establishment of the National Rural Health Mission (NRHM), by the government of India, in April 2005 expanded the network of public health facilities to many poor and disadvantaged households in rural areas [4]. However, the utilization of public health facilities is abysmally low and is marked by substantial heterogeneity across regions and socio-demographic groups [5]. In spite of a special focus on maternal and child health issues, the institutional delivery rate is less than 60% in 170 districts [6]. Increasing institutional delivery rates has been a key strategy for achieving Millennium Development Goal 5 of reducing maternal mortality, and was named by the World Health Organization as the “single most important factor in preventing maternal death” [7].

Given that the provision of health services is strongly related to improved population health, it is of paramount importance to understand the many factors that affect both the utilization and provision of health care. These factors operate on both the demand and supply side [8], which can be explained in a multi-level framework described by previous studies. The framework identifies three levels of barriers: the community and household level, the health service delivery level, and the health sector policy and strategic management level [9]. At the community and household level, the use of health services is limited by physical, financial, and social barriers, which are also known as demand-side barriers. Income, distance to the health facility, and socioeconomic characteristics influence the use of healthcare by Indian households [10]–[12]. Supply side barriers operate at the service delivery level. They are characteristics of the health system that exist outside the control of potential health service users and hinder uptake by individuals, households, or the community [13], [14]. Finally, both supply and demand side barriers can be addressed at the policy and strategic management level, where policy makers require reliable information to make informed decisions.

Previous studies have identified numerous demand-side factors as important barriers to healthcare utilization in developing countries, but surprisingly few have truly addressed the supply-side barriers [15]. While many studies have described severe shortages of essential supplies, medications, and human resources across countries including India, there is a gap in knowledge as to how these observed supply-side bottlenecks affect service provision [16], [17]. The few existing studies addressing how supply-side barriers affect uptake of reproductive health services have found large but inconsistent effects, urging further research. A study in Egypt found that facility quality variables were more influential in the uptake of intrauterine devices at public health facilities than demographic or geographic characteristics [18]. A similar study in Uganda identified several infrastructural and service characteristics, including running water, electricity, and staff accommodations, to be strongly associated with increased deliveries [19]. However, in a study conducted in South Africa, few facility characteristics were found to be associated with contraceptive adoption, though human resource availability was correlated with choice of contraception methods [20].

Quantifying how the availability of doctors, drugs, medical equipment, infrastructure, and staff training affects the provision of health services is of tremendous policy relevance because the government controls and distributes the resources to equip its health facilities, and because of its potential interactions with demand-side factors. In recent years, many countries have abolished user fees or provided financial incentives for seeking maternity-care, but whether health facilities are adequately equipped with the resources to meet the increased demand due to these policy measures is not well understood. For instance, an evaluation of the Indian conditional cash transfer program for pregnant women, Janani Suraksha Yojana (JSY), found that while the volume of deliveries increased, facilities also faced shortages of drugs and equipment [21]. An equally important question is to what extent the alleviation of these constraints would result in improved utilization of health services, and how these constraints should be prioritized. Using a large cross-sectional facility-level dataset, this study contributes to the research on supply-side factors by assessing the effect of facility-level indicators on the volume of deliveries conducted at public health facilities in India.

## Methodology

### 1. Health care system in India

The Indian public healthcare delivery system operates at four hierarchical levels: sub-centers (SC), primary health centers (PHC), community health centers (CHC), and district hospitals (DH). SC is the lowest level of the health system while DH is the highest level. Each district has one DH, which is well-equipped to handle complicated cases. At the next level, CHCs are the most endowed in terms of medical staff and equipment, followed by PHCs, and subsequently SCs, which are the peripheral contact point between the primary health care system and the community. In terms of coverage, CHCs serve a population of 80,000 to 120,000; PHCs cater to a population of 30,000, and SCs look after the needs of approximately 5,000 [22]. We focus our study on PHCs because they are a crucial component of care as the first point of contact between an individual and a qualified public-sector doctor, particularly for rural populations. Furthermore, in many states, PHCs have recorded a four-fold increase in in-facility deliveries, while other public and private health clinics have showed a decline since the implementation of the NRHM and JSY in 2005–2006 [23]. Therefore, we set out to perform the analysis only on the sample of PHCs.

At the time of data collection in 2007–08, a PHC was expected to have one medical officer and 16 paramedical and other staff, and to act as a referral unit for five to six SCs. In terms of infrastructure, PHCs are stipulated to have four to six inpatient beds. The activities of the PHCs involve curative, preventive, and promotional health care. PHCs are expected to be equipped to provide 24/7 normal and assisted deliveries, ante-natal care, post-natal care, newborn care, family planning, and full child immunizations [22].

### 2. Study hypotheses

The analytical approach in this study was developed to assess the relationship between supply-side barriers and provision of institutional delivery services in India. We hypothesized that facilities with adequate resources will perform a greater number of deliveries, vaccinate more children, and treat more illness. In this study, we specifically examined how the availability of health personnel, medical equipment, drugs, and other infrastructure, such as electricity and water, affected the volume of delivery at the PHCs in India.

### 3. Data source

We used health facility data from the third wave of the District Level Households Survey (DLHS-3) in India, collected during 2007–2008. The DLHS data are publicly available from the International Institute for Population Sciences, Mumbai [22]. The health facility survey in DLHS-3 collected information on resources available at the facilities and the volume of services delivered. In total, 18,068 sub-centers, 8,619 primary health centers, 4,162 community health centers, and 594 district hospitals were surveyed. We used information from the sample of 8,619 PHCs to assess the association between infrastructure availability and delivery of maternity-care. The PHC facility survey collected information on infrastructure availability (number of beds, rooms etc.), health personnel, medical equipment, availability of drugs, and existence of quality and training measures. Of the 8,619 PHCs surveyed, 95% were in rural areas. Per the DLHS-3 report, 76% of the PHCs had a medical officer, and less than a quarter (24%) had a female medical officer [11]. The sampled PHCs in the DLHS-3 survey reported serving an average population of 49,193 against the population norm of 30,000 in the plain areas [22].

### 4. Dependent variable

The main dependent variable analyzed in this study is the number of deliveries performed in each facility in the month before the survey. This is a count variable ranging from zero to 414 deliveries. About 27% of the facilities reported performing no deliveries in the prior month. The average number of deliveries per facility was 21.

### 5. Facility and district-level covariates/predictor variables

We included several covariates (Table 1) as predictors of the volume of facility-based deliveries, which are potentially important bottlenecks for provision of maternity-care. We divided these bottlenecks into six categories: (a) health personnel, (b) drug availability, (c) medical equipment availability, (d) infrastructure, (e) quality, and (f) demographic variables.

**Table 1:** Definition and descriptive statistics of variables, n = 8227.

	Variable	Definitions	Mean/percent	SD	Min	Max
	Dependent variable					
1	PHC Delivery	Count variable indicating number of deliveries performed at the primary health center in the month before the survey	20.83	42.81	0	414
	**Independent Variables**					
2	Doctor	Continuous variable that indicates the number of types of doctors/medical officers at the facility	1.49	0.88	0	4
3	Paramedic staffs	Continuous variable that indicates the number of paramedic staff at the facility	4.02	1.71	0	7
4	Availability of obstetric drug	Binary variable, coded as one if essential obstetric care drugs are available	62%	0.49	0	1
5	Availability of adequatenumber of beds	Binary variable, coded as one if the facility has more than minimum number of beds (4). Each PHC is mandated to have at least 4 beds.	68%	0.47	0	1
6	Availability of labor room	Binary variable, coded as one if labor room is available at the facility for delivery purpose	69%	0.46	0	1
7	Availability of obstetric and new-born equipment	Number of obstetric-care equipment available at the facility	1.28	1.07	0	4
8	Availability of communicationinfrastructure	Number of communication equipment, such as computer, telephone, internet, vehicle etc. available at the facility	1.50	1.14	0	6
9	Opening hours	Whether facility is opened 24 hours	53%	0.50	0	1
10	Electricity	Binary variable, coded as one if the facility has access to regular power supply/generators/invertors	62%	0.49	0	1
11	Rural	Facility location, rural is coded as one	95%	0.22	0	1
12	Rogi Kalyan Samiti (RKS)	Establishment of RKS at the facility is coded as one	75%	0.43	0	1
13	Staff training	Binary variable, coded as one if facility's staffs were trained in BEmOC or Skilled Birth Delivery	31%	0.46	0	1
14	Years of schooling	Continuous variable indicating the average years of school completed by adults over age 18 in the district	5.38	1.44	1.57	9.53
15	Percent in lowest wealth quintile	Percent of households in the district categorized as being in the lowest wealth quintile, based on assets	19%	0.17	0	85
16	Percent insured	Percent of households in the district with any member covered by a health scheme or health insurance	5%	0.07	0	49
17	Percent Hindu	Percent of households in the district where the head of household is Hindu	82%	0.22	0.001	100
18	Fertility rate	Average live births per person-year among ever married women, between January 1 2004 and date of data collection (2008)	0.09	0.03	0.01	0.20
19	Log catchment population	Natural log of the catchment population reported by the PHC	10.31	0.95	1.95	13.44

The DLHS-3 records human resources as a binary indicator for each type of position filled. We included a count variable for the number of types of medical officers at the PHC (male medical officer, female medical officer, AYUSH medical officer, contractual medical officer), and a separate variable which included the number of paramedical position types filled (staff nurse, pharmacist, female health worker, male health assistant, lab technicians, auxiliary nurse midwife, and additional staff nurse). To capture the availability of drugs at the health facilities, we used a binary variable from the survey that indicates if essential obstetric care drugs were available at the time of the survey. To measure the availability of medical equipment, we created a continuous index which measured the availability of delivery-specific equipment at the facility. The index ranged from 0 to 4, with facilities receiving 1 point for having each of the following items: normal delivery kit, equipment for assisted vacuum delivery, equipment for assisted forceps delivery, and equipment for manual vacuum aspiration. We also included a binary indicator for the availability of an adequate number of beds. Since the government of India set a norm of four to six beds per PHC, we defined four beds as an adequate number [24]. The general infrastructure variables included a count of the types of communication and transport equipment available (internet, telephone, computer, NIC terminal, and vehicles), whether the facility was open 24 hours per day, and whether the facility had access to electricity and/or a generator. Two additional variables were also included to capture facility quality and to explore if high quality facilities were more likely to produce higher volume of service. The first variable was establishment of Rogi Kalyan Samiti (RKS), also known as a Patient Welfare Committee, which is a facility-based management structure that aims to provide quality health care services by engaging the local population in the decision-making process. Secondly, an indicator of staff quality was captured by a binary indicator of Basic Emergency and Obstetric Care (BEmOC) or Skilled Birth Attendance (SBA) training received by staff in five years before the survey.

Though the focus of this study is to assess the effect of supply-side factors on maternity-care, we also included variables that might affect the demand for health services, such as socioeconomic, demographic, and geographic factors. In all models, we included the log of the facility's reported catchment area and a district-level fertility indicator as the most basic controls for demand. The district-level annual fertility rate for 2004–2008 was calculated among 15–49 year old women using the DLHS-3 ever married women questionnaire. Additional district-level demographic controls (included in only the final model) were calculated using the DLHS-3 household questionnaire. This included the average years of education among those over age 18; the percentage of households in the lowest wealth quintile (as defined by an asset score calculated by DLHS-3); the percentage of households with any member covered under a health plan or insurance; and the percentage of households where the head of household was Hindu. Sample weights were applied for all district-level calculations.

### 6. Statistical analysis

We used a negative binomial regression model to examine the association between the availability of resources at the health facility and the volume of deliveries. The negative binomial regression model is commonly used when the dependent variable is non-negative count data [25], [26]. We ruled out a Poisson regression model because of its strict assumption that the dependent variable has the same mean and variance µ_ig_ = exp(X_ig_ β_g_). The distribution of institutional deliveries in our dataset clearly exhibited over-dispersion, with a mean of 21 and a variance of 1832. A Poisson regression model would produce inefficient estimates in this case. Therefore, we preferred a negative binomial model over a Poisson model because it does not require the assumption of equality of the conditional mean and variance, and allows for unmeasured characteristics that generate over-dispersion in the count data [27].

We estimated the following negative binomial regression model:
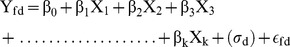
(1)where Y represents the outcome variable, the number of deliveries performed at the facility in the previous month, at the facility *f* in district *d*, and X_n_ represents the facility and district-level demographic factors affecting this outcome. *σ_d_* is the random effect that varies at the district level (Z_d_ ∼N(0, σ_d_)). The random intercept *σ*_d_ captures the effect of latent district-specific (time-invariant) covariates that cause some districts to have facilities that produce a greater volume of services than others, such as fewer private clinics or socio-demographic characteristics of the population that potentially affect the demand for facility-based delivery. We used Stata (version 13.0) for the analysis.

## Results

After excluding facilities with missing information on the variables used in this study, our analytical sample comprises 8,227 PHCs with approximately 200,000 births recorded in the month prior to the survey.

Table 1 displays the definitions and summary statistics for the dependent and independent variables. In row 1, the sample mean and standard deviation of the dependent variable are reported. The mean of the dependent variable, the number of deliveries, is significantly different from the variance justifying the choice of a negative binomial model over a Poisson model, since the dependent variable is widely dispersed. The summary statistics for the independent variables convey the landscape of resource availability. On average, facilities had 1.49 of the 4 medical officer types, and four types of paramedical staff. Nearly 62% of the PHCs had the essential obstetric care drugs, 68% had an adequate number of beds, and 69% of facilities had a separate labor room to provide delivery services to women. Having a separate labor room was neither sufficient nor necessary for conducting deliveries; 44% of facilities without a delivery room still reported a delivery in the last month. Conversely, 36% of facilities without any deliveries had a separate labor room. Slightly less than half of the PHCs (47%) provided services 24 hours per day, despite the guideline that PHCs should be open at all hours of the day. Approximately 62% had a regular supply of electricity available. Nearly all the facilities were rural (95%) and 75% of them had established RKS or a Patient Welfare Committee. While exploring the quality of staff, data suggested that only 31% of the facilities had personnel that were trained either in BEmOC or SBA. There was substantial variation in the district-level demographic characteristics. The percent of households in the lowest wealth quintile ranged from 0 to 85%, and the average years of schooling ranged from less than 2 to over 9 (mean 5.38 years). On average, 5% of households in a district included an individual covered by insurance, but the figure was as high as 49% in one district. Hinduism was the dominant religion in most districts (85% of households on average), but ranged from 0 to 100%.

The results from the negative binomial regression model are reported in Tables 2 and 3. Both tables report the incidence rate ratios (IRR) and 95% confidence interval (CI). An IRR greater than 1 implies that an increase in the independent variable is associated with an increase in the outcome variable and vice versa. Variables are grouped under the six previously described categories: health personnel, drug availability, equipment, infrastructure, facility quality, and district-level socio-demographic composition. In the first four columns of Table 2, groups of variables are included individually, controlling only for the catchment area's population and fertility rate. In column 5, we included all facility resource variables together. Results in column 1 highlight the importance of health personnel. A greater availability of medical and paramedical staff is statistically associated with an increase in the incidence rate of delivery by 1.08 [95% CI: 1.02–1.12] and 1.18 [95% CI: 1.14–1.21], respectively. Results from the other separate models indicate that the delivery rate is higher if essential obstetric drugs and medical equipment are available in the clinics, and increases substantially with the presence of a labor room (IRR: 1.89 [95% CI: 1.68–2.12]), adequate beds (IRR: 1.45 [95% CI: 1.29–1.63]), and 24 hours of operation (IRR: 1.65 [95% CI: 1.56–1.80]). Access to electricity and availability of communication infrastructures are also significantly associated with a higher volume of delivery. Column 5 shows that upon including all predictors in one model, the magnitudes of the effects are reduced, but the significance is maintained. The exception is the number of medical staff, which becomes insignificant. In this combined model, the facility predictors with the largest coefficients are availability of labor room (IRR: 1.53 [95% CI: 1.37–1.72]) and facility opening hours (IRR: 1.39 [95% CI: 1.28–1.52]). Fertility rate and population size are also positively correlated with the number of deliveries conducted at the PHC.

**Table 2:** Facility-level determinants of institutional deliveries in India.

	Outcome: Number of deliveries performed at the facility									
	Incidence Rate Ratios (IRR) (95% CI)									
	(1)		(2)		(3)		(4)		(5)	
***Health Personnel***										
Number of medical staffs	1.086**	[1.020,1.156]							1.028	[0.973,1.085]
Number of paramedic staffs	1.177**	[1.142,1.214]							1.075**	[1.042,1.108]
***Drug Availability***										
Essential obstetric drugs			1.499**	[1.356,1.657]					1.139**	[1.033,1.255]
***Equipment***										
Adequate beds					1.454**	[1.293,1.636]			1.196**	[1.075,1.331]
Availability of labor room					1.887**	[1.678,2.122]			1.533**	[1.369,1.717]
Adequate obstetric equipment					1.178**	[1.124,1.235]			1.080**	[1.035,1.128]
***Infrastructure***										
Communication infrastructure (phone, computer, internet)							1.357**	[1.300,1.416]	1.257**	[1.204,1.313]
Facility open 24 hours							1.651**	[1.516,1.799]	1.392**	[1.275,1.518]
Access to electricity/generator/invertor							1.293**	[1.179,1.418]	1.172**	[1.060,1.295]
Log catchment population	1.654**	[1.525,1.794]	1.699**	[1.523,1.895]	1.653**	[1.517,1.801]	1.546**	[1.447,1.651]	1.503**	[1.405,1.607]
Fertility rate	1.162**	[1.107,1.220]	1.126**	[1.067,1.188]	1.144**	[1.087,1.205]	1.148**	[1.095,1.204]	1.220**	[1.161,1.282]

(**p<0.001).

Table 3 reports the results from the models that additionally control for facility and staff quality, whether the facility is located in a rural area, and socio-demographic composition of the district. The socio-demographic variables include average adult education level in the district, percentage of population with health insurance, Hindu population, and percentage of population that is poor. For comparison, column 1 reports the results from the last column of Table 2. Column 2 includes indicator variables for location of the facilities (rural/urban), and facility and staff quality. Column 3 shows results from the random-effect model with all the facility variables. Finally, column 4 displays results from a random-effect model that additionally includes socio-demographic controls. In all models, the IRR for rural is less than 1 suggesting that rural PHCs are producing a lower volume of delivery service than urban PHCs, even when adjusting for catchment population size and other demand factors (IRR: 0.88 [95% CI: 0.80–0.97] in column 4). In the full model, including socio-demographic factors and the facility and staff quality variables, RKS is not significantly associated with the outcome, while staff's quality (BEmOC or SBA training) is significantly associated with the volume of delivery services (IRR: 1.05 [95% CI: 1.00–1.11]). Compared to column 1, inclusion of district-level demographic characteristics and random-effects alters the magnitude of several facility-level predictors, such as the drug, equipment, and infrastructure variables, but does not change the directionality or significance substantially. The exception is the number of medical staff, which attains significance in the final model. Most of the other explanatory variables have the expected signs and are statistically significant. As expected, the incidence-rate of delivery in a health facility with a labor room is higher than a facility without a labor room (IRR: 1.81 [95% CI: 1.68–1.95]). It is comforting to note that the main results do not change substantially across models, even after including district-level random-effect, meaning results are not a product of the observed district demographic characteristics, or unobserved factors captured in the random effect.

**Table 3:** Facility-level determinants of institutional deliveries in India.

	Outcome: Number of deliveries performed at the facility							
	Incidence Rate Ratios (IRR) (95% CI)							
	(1)		(2)		(3)		(4)	
***Health Personnel***								
Number of medical staffs	1.028	[0.973,1.085]	1.014	[0.962,1.069]	1.033*	[1.002,1.064]	1.046**	[1.014,1.078]
Number of paramedic staffs	1.075**	[1.042,1.108]	1.071**	[1.039,1.105]	1.054**	[1.036,1.072]	1.058**	[1.039,1.076]
***Drug Availability***								
Essential obstetric drugs	1.139**	[1.033,1.255]	1.147**	[1.042,1.261]	1.184**	[1.120,1.251]	1.169**	[1.107,1.235]
***Equipment***								
Adequate beds	1.196**	[1.075,1.331]	1.204**	[1.083,1.338]	1.247**	[1.168,1.331]	1.245**	[1.167,1.329]
Availability of labor room	1.533**	[1.369,1.717]	1.535**	[1.373,1.716]	1.854**	[1.724,1.994]	1.811**	[1.684,1.946]
Adequate obstetric equipment	1.080**	[1.035,1.128]	1.076**	[1.032,1.122]	1.097**	[1.072,1.122]	1.092**	[1.067,1.116]
***Infrastructure***								
Communication infrastructure (phone, computer, internet)	1.257**	[1.204,1.313]	1.257**	[1.205,1.312]	1.076**	[1.049,1.103]	1.076**	[1.049,1.104]
Facility open 24 hours	1.392**	[1.275,1.518]	1.379**	[1.266,1.503]	1.435**	[1.358,1.515]	1.429**	[1.353,1.509]
Access to electricity/generator/invertor	1.172**	[1.060,1.295]	1.161**	[1.050,1.284]	1.085**	[1.024,1.150]	1.117**	[1.053,1.183]
***Quality variables***								
Rogi Kalyan Samiti (RKS)			0.981	[0.875,1.099]	1.052	[0.989,1.120]	1.060	[0.996,1.127]
Staff training			1.123*	[1.026,1.228]	1.045	[0.994,1.098]	1.052*	[1.001,1.105]
Rural			0.773*	[0.633,0.945]	0.891*	[0.811,0.979]	0.880**	[0.801,0.966]
Average years of school among adults							0.844**	[0.817,0.872]
% population in lowest wealth quintile							0.472**	[0.356,0.624]
% population insured							1.03	[0.632,1.678]
% Hindu population							1.354**	[1.139,1.610]
log catchment population	1.503**	[1.405,1.607]	1.502**	[1.409,1.602]	1.144**	[1.108,1.181]	1.129**	[1.094,1.166]
Fertility rate	1.220**	[1.161,1.282]	1.215**	[1.158,1.276]	1.084**	[1.044,1.125]	1.062**	[1.019,1.106]
District random-effects					X		X	

(*p<0.05; **p<0.01).

It is quite possible that availability of labor room might confound the effect of other variables because some of these variables could be largely dependent on the pre-requisite of having a labor room. In theory, presence of a labor room is a pre-requisite to conduct delivery in public facilities, however our analytical data portrays a contrasting picture. In our sample of 8227 facilities, approximately 2591 facilities conducted delivery without a labor room indicating that about 32% of the facilities are providing delivery-care without having access to separate labor room. Nonetheless, as a robustness check, we reestimate eq(1) separately for facilities with and without a labor room to purge out the confounding association between delivery room and other facility characteristics. Results are reported in Table 4. Results are qualitatively similar to Table 3. Availability of paramedical staffs and essential obstetric drugs continues to be significant predictor of delivery-care regardless of availability of a labor room. Availability of beds and obstetric equipment has a larger effect in facilities without a labor room compared to facilities with a labor room. Facility opening hours remain significantly associated with delivery-care in both types of facilities.

**Table 4:** Facility-level determinants of institutional deliveries in India.

	Outcome: Number of deliveries performed at the facility			
	Incidence Rate Ratios (IRR) (95% CI)			
	Facilities without labor room (N = 2591)		Facilities with labor room (N = 5636)	
	(1)		(2)	
***Health Personnel***				
Number of medical staffs	0.96	[0.888,1.038]	1.069**	[1.034,1.105]
Number of paramedic staffs	1.087**	[1.041,1.135]	1.055**	[1.035,1.075]
***Drug Availability***				
Essential obstetric drugs	1.287**	[1.135,1.458]	1.116**	[1.052,1.185]
***Equipment***				
Adequate beds	1.328**	[1.170,1.508]	1.155**	[1.074,1.243]
Adequate obstetric equipment	1.297**	[1.225,1.373]	1.050**	[1.025,1.076]
***Infrastructure***				
Communication infrastructure (phone, computer, internet)	1.148**	[1.072,1.229]	1.068**	[1.039,1.098]
Facility open 24 hours	1.826**	[1.608,2.073]	1.361**	[1.283,1.443]
Access to electricity/generator/invertor	0.986	[0.860,1.130]	1.150**	[1.079,1.226]
***Quality variables***				
Rogi Kalyan Samiti (RKS)	1.086	[0.826,1.429]	0.845**	[0.765,0.932]
Staff training	1.151*	[1.004,1.320]	1.021	[0.954,1.094]
Rural	0.897	[0.780,1.032]	1.101**	[1.044,1.161]
Average years of school among adults	0.730**	[0.682,0.782]	0.888**	[0.857,0.921]
% population in lowest wealth quintile	0.173**	[0.0970,0.307]	0.699*	[0.512,0.954]
% population insured	1.06	[0.255,4.398]	1.155	[0.693,1.925]
% Hindu population	2.046**	[1.467,2.855]	1.364**	[1.130,1.646]
log catchment population	1.088*	[1.012,1.171]	1.173**	[1.131,1.216]
Fertility rate	1.131**	[1.040,1.229]	1.059*	[1.011,1.110]
District random-effects	X		X	

(*p<0.05; **p<0.01).

## Discussion

Despite targeted efforts by government and multinational agencies, close to one-third of the deliveries in India occur at home without any medical supervision. There are many factors that hinder utilization of maternity-care in India. For example, past nationally representative surveys have reported cost to be an important barrier to facility-based delivery and an evaluation of JSY found a significant effect on institutional births in India [22], [28]. Similarly, several other studies have identified socioeconomic, demographic, and geographic barriers to an institutional use of maternal-care in India and showed a negative relationship between distance to health facility and in-facility birth in India [8], [9], [10], [29], [30]. The majority of the factors examined in these studies are demand-side constraints that mainly operate at the household level. Though it is generally hypothesized that supply-side factors at the facility level have a strong impact on provision and utilization of health services, there exists a surprising scarcity of systematic attempts to understand the association between facility-level bottlenecks and skilled delivery.

Our study explores the association between facility characteristics and the volume of in-facility deliveries performed in India, taking the facility as the unit of analysis. Our main results suggest that availability of labor rooms, opening hours of the facility, and adequacy of general medical equipment and infrastructure are the primary facility-level drivers of institutional delivery at PHCs in India. In contrast, community involvement through RKS, recent relevant staff training, and greater staff numbers had weak or low-magnitude associations. These results are robust to inclusion of district-level socio-demographic factors and a district random effect, indicating that they cannot be explained simply by inter-district variations.

The study has important policy implications because the government has the capabilities and resources to make changes to its health facilities. This is in contrast to efforts of impacting individual-level demand factors, which may require years of complex economic and social change. Our results indicate that equipping more PHCs with labor rooms, and making them meet the requirement of 24/7 service would be important steps to achieving more facility-based deliveries.

The descriptive results of our study also highlight that there is a great deal of room for relevant improvements in these areas. Specifically, only slightly more than half of facilities were open 24 hours a day, and 30% of facilities were not equipped with labor rooms. Beyond the issue of encouraging patients to seek care is the quality and safety of services available at the facility for those who do choose to come. For instance, 625 facilities reported conducting deliveries in the last month despite not having any of the four pieces of equipment available, including a basic delivery kit.

RKS is a strategy to improve the quality of management responses and thereby, facilitate the strengthening of health systems as well as health outcomes. RKS has been an important step under NRHM to increase community participation in the management of the health facilities. However, our results indicate that decentralized decision-making by RKS does not have a strong effect on the provision of delivery care at PHC level. The DLHS-3 report finds that constitution and utilization of untied RKS funds in the CHC and DH have been successfully implemented. However, the implementation of RKS proved problematic at the PHC level. About 70% of the PHC did not spend the RKS untied funds and very few facilities displayed the citizen's charter. Some studies have revealed that inadequate support systems for capacity building and training are constraints which weaken the impact of RKS [31]. This study also reports an absence of regular meeting of stakeholders and autocratic decision-making at many PHCs, thereby diluting the purpose of establishing RKS. Therefore, for RKS to attract users and improve quality of health services, the overall functioning needs to be strengthen at the PHC level by creating a proper grievance redressal system, conducting regular meetings with the stakeholders, and capacity building.

Our study has several strengths. It contributes to the scant literature on the effect of facility characteristics on institutional delivery in India. Given that many births in India are unsafe and home-based, it is important to understand what improvements can be made at the facility level to increase in-facility deliveries. The use of nationally representative data is a strength of this study, and our findings are reliable due to the large sample size. Estimation of a count model, specifically a negative binomial regression model, adds to the strength of this study because a count model is the correct model to apply with this type of outcome variable.

However, our study is not free from limitations. First and foremost, the study is based on cross-sectional data and therefore, causality cannot be inferred. Our analysis may suffer from reverse causality. It is possible that facilities conducting more deliveries are likely to receive more resources or vice-versa. Given the cross-sectional nature of our dataset, pinning down the direction of causality is not possible. A panel data is needed to address the issue of reverse causality or temporality. The dependent variable, the number of deliveries performed at the facility, could suffer from measurement bias as the data quality depends on the specific data management system at each facility. We also do not include activities related to in-home skilled birth attendants, which has been an alternative solution proposed for safe deliveries. Several of the independent variables of interest could also improve in terms of measurement. For example, DLHS-3 only captured a binary indicator for whether each type of personnel was present. Although the PHC standards call for only one individual in each type of position, it would be preferable to capture the total number of personnel working at the facility in case this quota is ever exceeded. It would also be preferable to include a measure of total fertility rather than looking within ever-married women. This was not possible with DLHS-3 data because fertility questions were only asked of ever-married women, and district-level estimates for the time period of interest were not available from other sources.

In India, the private sector plays an important role in delivering healthcare. Due to poor quality of healthcare in public hospitals, many households prefer to visit private clinics to seek maternity-care. Per DLHS 2007-2008, about 17% of the institutional deliveries were performed at private health clinics. However, due to unavailability of data on private facilities, we could not include private clinics/hospitals in our study. But, from a policy perspective, it is more relevant to analyze only public facilities as it is in this sector where government can intervene and improve the quality of healthcare provision because government has limited control over private sector resource allocation.

While we have chosen to focus on the PHC in our analysis, future research could be expanded to examine the role of supply-side factors in other levels of the health system, namely rural and district hospitals. Future studies should also further examine the interaction between supply and demand side factors, to tease out complicated relationships that exist between the two. We attempted to address this by including district-level demographic factors in our final model, but many factors, such as distance to the facility and income are better studied at the individual rather than aggregate level. The supply side factors found to be significant may also interact with demand factors by altering patient perceptions of facility quality, which has been shown to drive demand for health services [32]. Finally, it is important to incorporate the quality of care, mainly the behavior of the providers such as doctors, nurses, and supporting staffs. Unfortunately, our study could not establish the relationship between provider's quality and institutional delivery because the DLHS data did not provide information on quality of the providers. Therefore, future studies should attempt to include measures of quality of care to address concerns that increased volume could affect quality of care.
